# Supplementary material for: Disentangling dispersion from mean reveals true heterogeneity-diversity relationships
Source: Nat Commun. 2025 Sep 29;16:8532. doi: 10.1038/s41467-025-64287-0 (PMC12480598; doi:10.1038/s41467-025-64287-0)
Supplement: Supplementary file 1 — Supplementary Information [file 41467_2025_64287_MOESM1_ESM.pdf]

Supplementary Information

Disentangling dispersion from mean reveals true heterogeneity-diversity relationships

Cameron Pellett[1*] and Rubén Valbuena[1]

[1]Department of Forest Resource Management, Swedish University of Agricultural Sciences, Umeå, 901 83, Sweden.

*Corresponding author(s). E-mail(s): cameron.pellett@slu.se;

Supplementary Note 1. Determination of a variable's boundaries

Understanding a variable's boundaries is fundamental for the correct evaluation of the distribution's heterogeneity and the influence of that heterogeneity on other variables. In this section we aim to make determination of boundaries clear for variables not discussed in the main text. To do this, we give practical guidelines and highlight possible misconceptions with examples from numerous fields. A variable's boundaries limit the extremes of the variable, and can often arise from what can be considered a fundamental constants. A variables boundaries are *not* the observed minimum nor maximum value in empirical data. We start with the most trivial examples, then cover more complex cases and examples where boundaries may not need to be considered, and finally finish with discussing truncated variables. The broad set of examples is primarily given for developing intuition about the phenomenon of boundaries, and not necessarily intended to be used as reference.

The most common and trivial boundary is zero, *e.g.* trees cannot have a height lower than zero, the mass of a animal cannot be less than zero, *etc.* This boundary at zero is clearly distinct from the observed minimum in empirical data for the same examples, *i.e.* a tree will never be observed to have a height of zero. Lower-bounded

examples like these will often be described as being on the open interval, $(0, \infty)$, with a lower bound in the set of all real numbers and an upper bound not in the same set. Another common and trivial example is proportions with boundaries at zero and one, *e.g.* proportion of land covered by forest, proportion of government spending in a department, *etc.* Again, for these examples, these boundaries will often (but not always) be distinct from the observed minimum and maximum in empirical data. Double-bounded examples like these will often be described as being on the closed interval, [0,1], with both boundaries being in the set of all real numbers.

The above examples will cover a large proportion of use cases for δ. However, there are numerous other examples specific to individual fields of study. Some examples in biology of variables with upper bounds include replication rates of mammals or DNA, which are limited, respectively, by gestation even in an environment with abundant resources without competition, or by the elongation step when using polymerase chain reaction even if the process is without inhibitors [1]. These examples may be observed in empirical data in a controlled environment without other limiting factors, or found conceptually based on known limitations. Other examples include the maximum body size of insects due to limitations of oxygen concentration and diffusion in tracheal systems [2], the maximum speed of interactions and transfer of information at the speed of light [3], and biochemical depth limits for marine fish [4].

Variables without significant boundaries also exist, due to the majority of the distribution's density being located far from the boundaries, or due to the boundaries truly not existing. Examples of insignificant boundaries include the measurement of climatic temperature on Earth's surface, where the boundary at absolute zero (-273.15 °C; 0 Kelvin) will have no meaningful impact on the temperature distribution, and empirical observations on Earth will never be observed at or near the true boundary. Likewise, even some of the single-bounded examples already given may have additional, though insignificant, boundaries. For example, maximum plant and animal sizes may be limited by hydrology and mechanics, but for the example of tree sizes the mechanical upper limit is likely to be roughly 3 to 4 times greater than observed individuals [5]. Additionally, the lower depth boundary at 0 m may not be significant for deep sea fish, such as the hadal snailfish (*Notoliparis kermadecensis*; Liparidae) [4]. In these cases it is reasonable to disregard insignificant boundaries, particularly when there is some uncertainty around their exact value.

Truncated variables are a distinct category from those described above and in the main text. Truncated variables primarily arise due to measurement limitations, where individuals or units below or above a threshold do exist but are not observed. For example, foresters and forest ecologists often measure tree diameters at a fixed height of 1.3 meters above the ground. As a result, the diameter distribution has a distinct end point greater than the true boundary at zero. This arises because trees with smaller diameters than the observed end points are not tall enough to be measured. As a result, a distribution's mean can be moved towards the measurement threshold and a greater number of units will simply no longer be observed. In this case there may be little to no concentration at the threshold, assuming the true boundary is far from the measurement threshold. We consider this to be a measurement problem specific to individual fields of study, so it is not possible to give universal solutions. It may be necessary and possible in some instances to model the unmeasured observations, such as assuming an underlying parametric distribution and sampling the remaining observations. However, this must be carried out with caution and often solutions to the underlying measurement problem may be necessary. Further research into each individual problem will be necessary.

Supplementary Note 2. The maximum variance is dependent on the mean for beta distributed variables

Here we give a proof that the maximum variance of a beta distributed variable with a given mean is

$$max_\mu(\sigma^2) = \mu(1-\mu)\,. \tag{51}$$

First note that the variance of a beta distributed variable continually increases as the parameters p and q approach their minimum at zero (Equation 12). The proof, thus, consists of finding the maximum variance by taking the limit as p goes to zero and q is relative to p by some constant, c ($p \to 0^+$ and $q = cp$). The constant, c, will clearly dictate the mean of the variable (Equation 7). Therefore, the aim is to find a limit that be expressed with c and then substitute the relationship between c and μ.

The maximum variance of a beta distributed variable can be given by the limit:

$$max_\mu(\sigma^2) = \lim_{p \to 0^+, q=cp} \frac{pq}{(p+q)^2(p+q+1)}\,, \tag{52}$$

However, evaluating this equation directly gives the indeterminate form 0/0. To evaluate this limit we use L'Hôpital's rule, which states that

$$\lim_{x\to c}\frac{f(x)}{g(x)} = \lim_{x\to c}\frac{\frac{d}{dx}f(x)}{\frac{d}{dx}g(x)}\,, \tag{53}$$

if evaluating the limit gives the indeterminate form 0/0. Using the rule we find

$$\begin{aligned}
max_{\mu}(\sigma^2) &= \lim_{p\to 0^+, q=cp}\frac{pcp}{(p+cp)^2(p+cp+1)} = \lim_{p\to 0^+, q=cp}\frac{\frac{d}{dp}[cp^2]}{\frac{d}{dp}[(c+1)^2p^2((c+1)p+1)]}\\
&= \lim_{p\to 0^+, q=cp}\frac{\frac{d}{dp}[2cp]}{\frac{d}{dp}[2p(c+1)^2((c+1)p+1)+(c+1)^2p^2(c+1)]}\\
&= \lim_{p\to 0^+, q=cp}\frac{2c}{2(c+1)^2((c+1)p+1)+2p(c+1)^2(c+1)+2p(c+1)^3}\\
&= \frac{2c}{2(c+1)^2(0+1)+0+0} = \frac{2c}{2(c+1)^2}\\
&= \frac{c}{(c+1)^2}
\end{aligned}$$

Now we relate c to the mean (μ). With Equation (7) we find that

$$q = \frac{1}{\mu}p - p = (\frac{1}{\mu} - 1)p\,. \tag{54}$$

Therefore, the constant c that relates p to q for a given μ is found with

$$c = \frac{1}{\mu} - 1\,. \tag{55}$$

All that remains is substituting Equation (55) for c and simplifying. This gives

$$\begin{aligned}
max_{\mu}(\sigma^2) &= \lim_{p\to 0^+, q=cp}[\sigma^2] = \frac{c}{(c+1)^2}\\
&= \frac{1/\mu - 1}{(1/\mu)^2} = \mu^2(1/\mu - 1) = \mu - \mu^2\\
&= \mu(1-\mu)\,.
\end{aligned} \tag{56}$$

Supplementary Figures

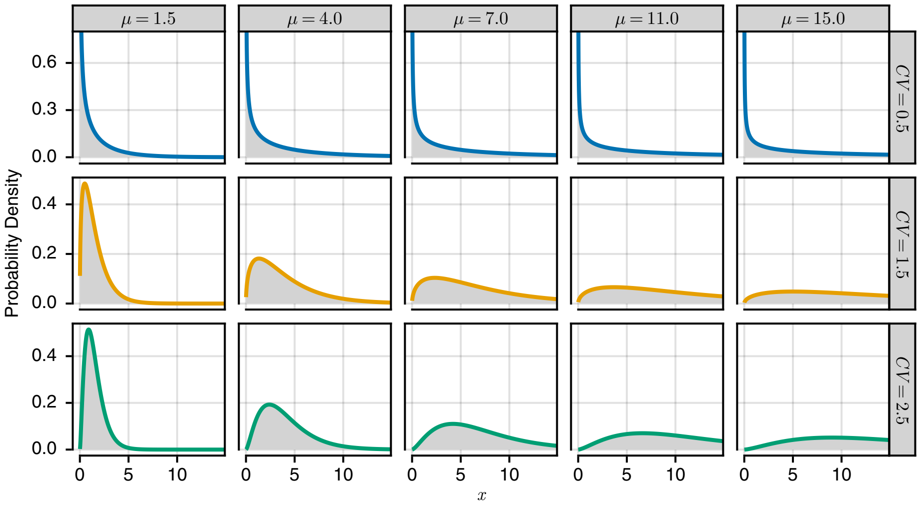

Supplementary Fig. S1 Gamma distributed variables with fixed coefficient of variation (CV), fixed skewness, and with a changing mean (μ). Note that this is equivalent to fixing the gamma distributions shape parameter (k; see Equation (24)) and modifying the mean solely with the scale parameter (δ_L) relying on the boundary minimising dispersion in one direction.

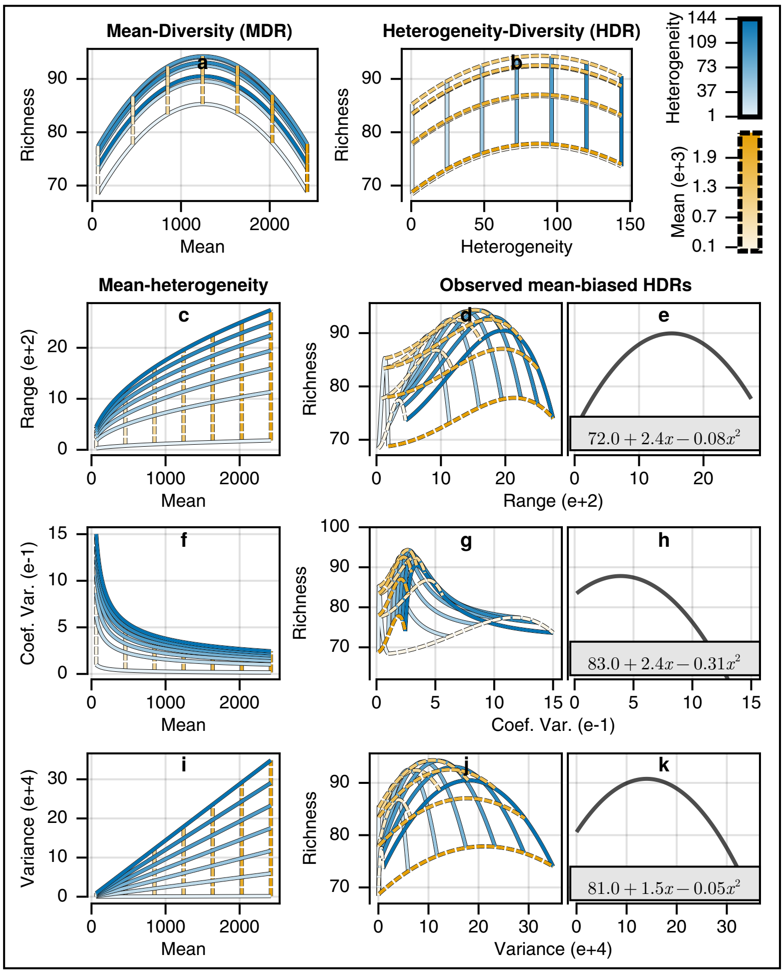

Supplementary Fig. S2 Retrieving true heterogeneity-diversity relationships (HDRs) with mean-independent measures of statistical dispersion for a variety of theoretical richness relationships (δ, eq. 1). The theoretical richness (D) model used in this figure is $D = 66.395 + 0.03 \cdot \mu - 1.2 \times 10^{-5} \cdot \mu^2 + 0.210 \cdot \delta - 1.2 \times 10^{-3} \cdot \delta^2$. Artifacts arose for all mean-biased heterogeneity measures, shown in panels **a-d**, **f-g**, and **i-j** with fixed levels of mean (orange lines) and heterogeneity (blue lines).

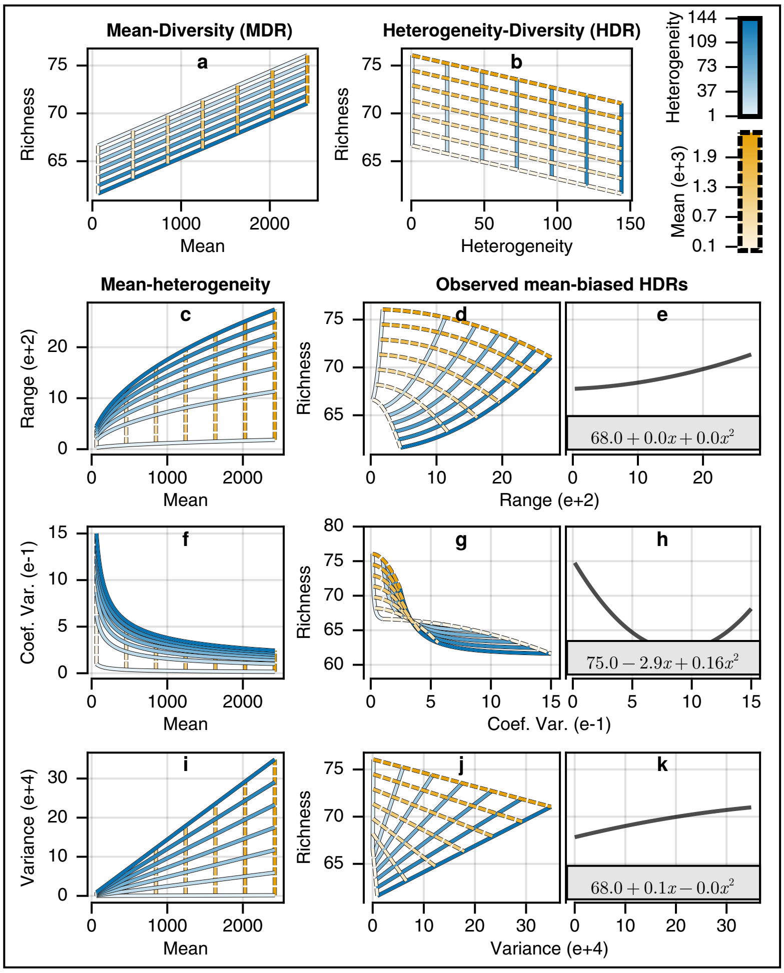

Supplementary Fig. S3 Retrieving true heterogeneity-diversity relationships (HDRs) with mean-independent measures of statistical dispersion for a variety of theoretical richness relationships (δ, eq. 1). The theoretical richness model used in this figure is $D = 66.395 + 0.004 \cdot \mu - 0.035 \cdot \delta$. Artifacts arose for all mean-biased heterogeneity measures, shown in panels **a-d**, **f-g**, and **i-j** with fixed levels of mean (orange lines) and heterogeneity (blue lines).

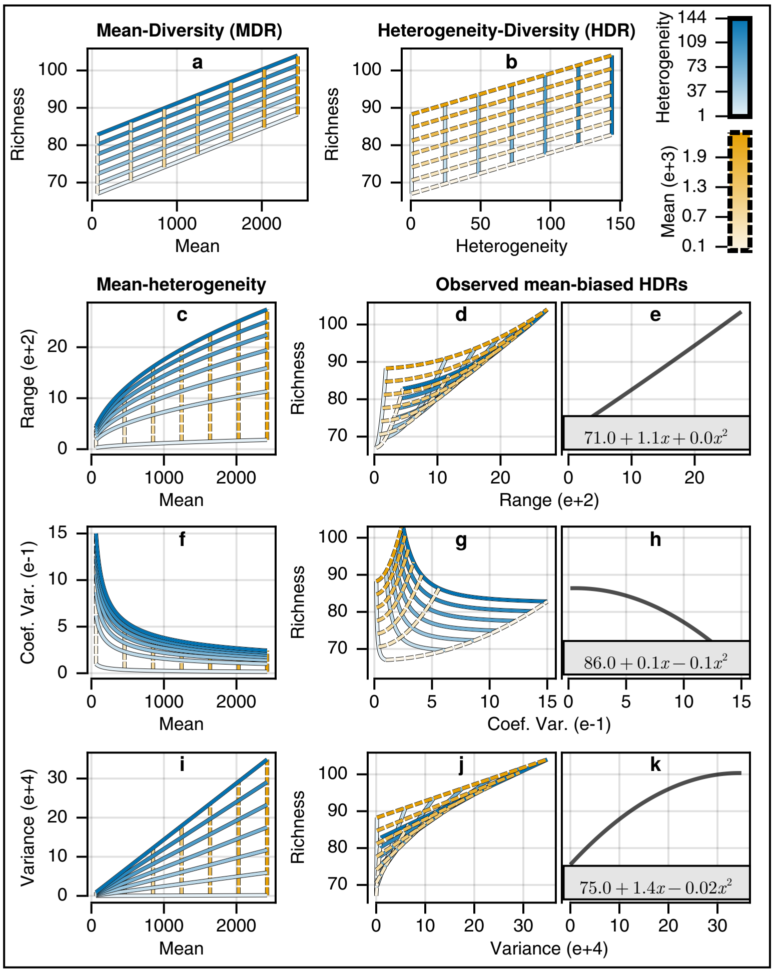

Supplementary Fig. S4 Retrieving true heterogeneity-diversity relationships (HDRs) with mean-independent measures of statistical dispersion for a variety of theoretical richness relationships (δ, eq. 1). The theoretical richness (D) model used in this figure is $D = 66.395 + 0.009 \cdot \mu + 0.110 \cdot \delta$. Artifacts arose for all mean-biased heterogeneity measures, shown in panels **a-d**, **f-g**, and **i-j** with fixed levels of mean (orange lines) and heterogeneity (blue lines).

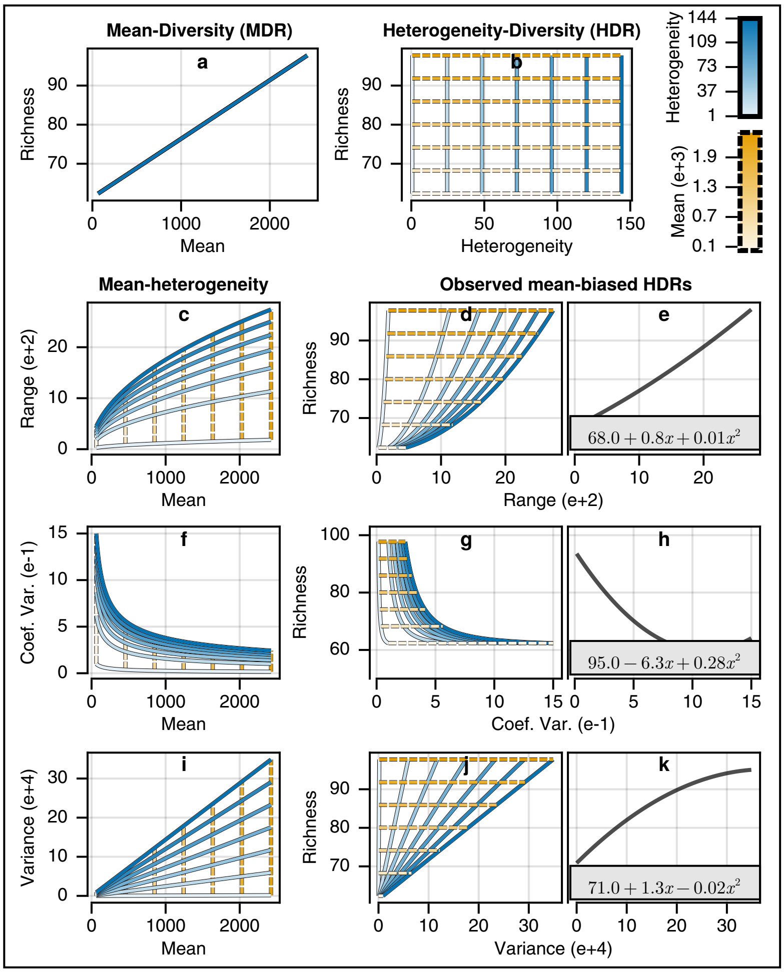

Supplementary Fig. S5 Retrieving true heterogeneity-diversity relationships (HDRs) with mean-independent measures of statistical dispersion for a variety of theoretical richness relationships (δ, eq. 1). The theoretical richness (D) model used in this figure is $D = 66.395 + 0.015 \cdot \mu$. Artifacts arose for all mean-biased heterogeneity measures, shown in panels **a-d**, **f-g**, and **i-j** with fixed levels of mean (orange lines) and heterogeneity (blue lines).

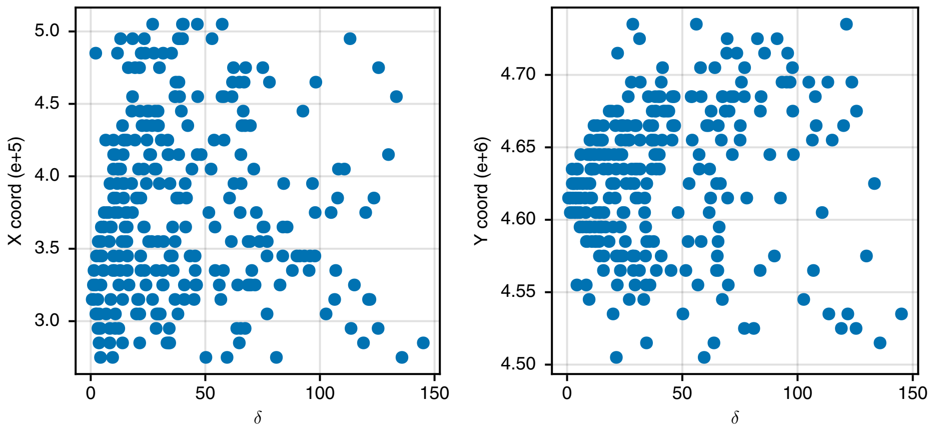

Supplementary Fig. S6 δ land elevation above sea level is not correlated with plot location. X and Y coordinates (coords.) are in UTM zone 31N projection (EPSG: 32631). Breeding bird species richness was measured for Catalonia by the Institut Català d'Ornitologia for 386 (n = 285 retained for analysis) 10x10 km continuous grid cells [6]. Land elevation above sea level was measured by the Shuttle Radar Topography Mission [7].

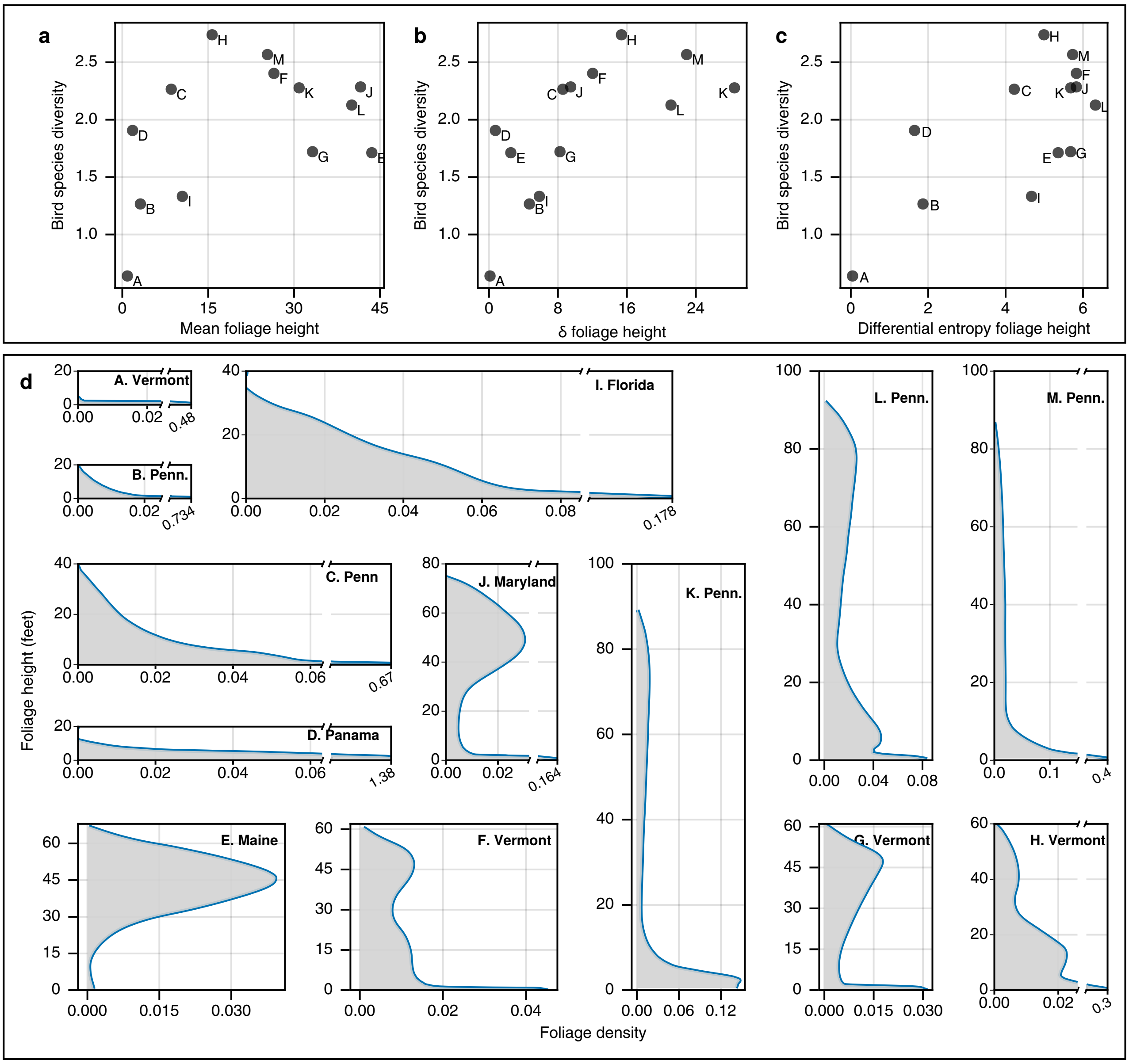

Supplementary Fig. S7 Reanalysis of foliage height and bird species diversity data from MacArthur and MacArthur [8]. **a-c,** Relationships between foliage structural measures and bird species diversity (Shannon diversity index of bird species abundances; n = 13). **d,** Digitised foliage density data from MacArthur and MacArthur [8]. Pearson's correlation coefficient was calculated for the relationship in panels **a-c,** with significant determined using a two-sided t-test (**a**: r = 0.43, t-statistic: 1.58, df = 11, p-value = 0.14; **b**: r = 0.67, t-test statistic: 2.98, df = 11, p-value = 0.0125; **c**: r = 0.71, t-test statistic: 3.32, df = 11, p-value = 0.0068).

References

[1] Madadelahi, M., Agarwal, R., Martinez-Chapa, S. O. & Madou, M. J. A roadmap to high-speed polymerase chain reaction (PCR): COVID-19 as a technology accelerator. *Biosensors and Bioelectronics* **246**, 115830 (2024). URL https://www.sciencedirect.com/science/article/pii/S0956566323007728.

[2] Kaiser, A. *et al.* Increase in tracheal investment with beetle size supports hypothesis of oxygen limitation on insect gigantism. *Proceedings of the National Academy of Sciences* **104**, 13198–13203 (2007). URL https://pnas.org/doi/full/10.1073/pnas.0611544104.

[3] Einstein, A. *The Principle Of Relativity, Ch. 3 On the electrodynamics of moving bodies* (1923). URL http://archive.org/details/in.ernet.dli.2015.214561.

[4] Yancey, P. H., Gerringer, M. E., Drazen, J. C., Rowden, A. A. & Jamieson, A. Marine fish may be biochemically constrained from inhabiting the deepest ocean depths. *Proceedings of the National Academy of Sciences* **111**, 4461–4465 (2014). URL https://www.pnas.org/doi/full/10.1073/pnas.1322003111. Publisher: Proceedings of the National Academy of Sciences.

[5] Kempes, C. P., Koehl, M. a. R. & West, G. B. The Scales That Limit: The Physical Boundaries of Evolution. *Frontiers in Ecology and Evolution* **7** (2019). URL https://www.frontiersin.orghttps://www.frontiersin.org/journals/ecology-and-evolution/articles/10.3389/fevo.2019.00242/full. Publisher: Frontiers.

[6] Estrada, J., Pedrocchi, V., Brotons, L. & Herrando, S. *Atles Dels Ocells Nidificants de Catalunya 1999–2002 [Breeding Birds Atlas of Catalonia].* (Institut Català d'Ornitologia/Lynx Editions, Barcelona, 2004).

[7] Jarvis, A., Reuter, H. I., Nelson, A. & Guevara, E. Hole-filled seamless SRTM data V4, International Centre for Tropical Agriculture (CIAT). (2008). Available from https://srtm.csi.cgiar.org (accessed 2024-03-19).

[8] MacArthur, R. H. & MacArthur, J. W. On Bird Species Diversity. *Ecology* **42**, 594–598 (1961). URL https://onlinelibrary.wiley.com/doi/abs/10.2307/1932254. Https://doi.org/10.2307/1932254.
