# Supplementary material for: Disentangling dispersion from mean reveals true heterogeneity-diversity relationships
Source: Nat Commun. 2025 Sep 29;16:8532. doi: 10.1038/s41467-025-64287-0 (PMC12480598; doi:10.1038/s41467-025-64287-0)
Supplement: Supplementary file 3 — Reporting Summary [file 41467_2025_64287_MOESM3_ESM.pdf]

nature portfolio

Corresponding author(s): Cameron Pellett

Last updated by author(s): Aug 25, 2025

Reporting Summary

Nature Portfolio wishes to improve the reproducibility of the work that we publish. This form provides structure for consistency and transparency in reporting. For further information on Nature Portfolio policies, see our Editorial Policies and the Editorial Policy Checklist.

Statistics

For all statistical analyses, confirm that the following items are present in the figure legend, table legend, main text, or Methods section.

n/a	Confirmed	
☐	☒	The exact sample size (*n*) for each experimental group/condition, given as a discrete number and unit of measurement
☐	☒	A statement on whether measurements were taken from distinct samples or whether the same sample was measured repeatedly
☐	☒	The statistical test(s) used AND whether they are one- or two-sided<br>*Only common tests should be described solely by name; describe more complex techniques in the Methods section.*
☐	☒	A description of all covariates tested
☐	☒	A description of any assumptions or corrections, such as tests of normality and adjustment for multiple comparisons
☐	☒	A full description of the statistical parameters including central tendency (e.g. means) or other basic estimates (e.g. regression coefficient) AND variation (e.g. standard deviation) or associated estimates of uncertainty (e.g. confidence intervals)
☐	☒	For null hypothesis testing, the test statistic (e.g. *F*, *t*, *r*) with confidence intervals, effect sizes, degrees of freedom and *P* value noted<br>*Give P values as exact values whenever suitable.*
☒	☐	For Bayesian analysis, information on the choice of priors and Markov chain Monte Carlo settings
☒	☐	For hierarchical and complex designs, identification of the appropriate level for tests and full reporting of outcomes
☐	☒	Estimates of effect sizes (e.g. Cohen's *d*, Pearson's *r*), indicating how they were calculated

Our web collection on statistics for biologists contains articles on many of the points above.

Software and code

Policy information about availability of computer code

Data collection	The Julia programing language (v1.10.4) was used to collect and process data. All code is available in the the public repository without restrictions found at https://zenodo.org/doi/10.5281/zenodo.11561447. The full list of 303 package dependencies is given in the Zenodo repository Manifest.toml
Data analysis	The Julia programing language (v1.10.4) was used to analyse data. All code is available in the the public repository without restrictions found at https://zenodo.org/doi/10.5281/zenodo.11561447. The full list of 303 package dependencies is given in the Zenodo repository Manifest.toml.

For manuscripts utilizing custom algorithms or software that are central to the research but not yet described in published literature, software must be made available to editors and reviewers. We strongly encourage code deposition in a community repository (e.g. GitHub). See the Nature Portfolio guidelines for submitting code & software for further information.

Data

Policy information about availability of data

All manuscripts must include a data availability statement. This statement should provide the following information, where applicable:

- Accession codes, unique identifiers, or web links for publicly available datasets
- A description of any restrictions on data availability
- For clinical datasets or third party data, please ensure that the statement adheres to our policy

The breeding bird species abundance data analysed in this study is available online in a Zenodo repository with no restrictions (https://doi.org/10.5281/

zenodo.11561447). The SRTM and Copernicus land cover data are available online with no restrictions in a CGIAR-CSI and Zenodo repository, respectively (https://srtm.csi.cgiar.org/, and https://doi.org/10.5281/zenodo.3939050, respectively). Source data are provided with this paper.

Research involving human participants, their data, or biological material

Policy information about studies with human participants or human data. See also policy information about sex, gender (identity/presentation), and sexual orientation and race, ethnicity and racism.

Reporting on sex and gender	not collected
Reporting on race, ethnicity, or other socially relevant groupings	not collected
Population characteristics	not collected
Recruitment	not collected
Ethics oversight	not collected

Note that full information on the approval of the study protocol must also be provided in the manuscript.

Field-specific reporting

Please select the one below that is the best fit for your research. If you are not sure, read the appropriate sections before making your selection.

☐ Life sciences ☐ Behavioural & social sciences ☒ Ecological, evolutionary & environmental sciences

For a reference copy of the document with all sections, see nature.com/documents/nr-reporting-summary-flat.pdf

Ecological, evolutionary & environmental sciences study design

All studies must disclose on these points even when the disclosure is negative.

Study description	The study was designed to evaluate if presently used measures of heterogeneity (e.g. variance, range, Gini, entropy) are biased by the mean and subsequently distort observed heterogeneity relationships with other variables, leading to the drawing of flawed ecological theory.
Research sample	There are three sample types in this study. Land elevation above sea level was measured by NASA's shuttle radar topography mission (SRTM) with a resolution of 3 arcsec. A mean-balanced sample of n = 101,490 60 x 60 arcsec transects were taken from the land elevation product for analysis. Crop cover percentage was predicted at a resolution of 3.57 arcsec by the the Copernicus Land Monitoring Service using vegetation data collected from the ESA PROBA-V satellite. A mean-balanced sample of n = 39,690 71.4 x 71.4 arcsec transects were taken from the crop cover product for analysis. Breeding bird species in Catalonia, Spain, sampled in two stages upscaled to n = 385 10 x 10 km UTM squares. Land elevation transects (from the SRTM) were taken for the same n = 385 10 x 10 km UTM squares.
Sampling strategy	Two-phase stratified random sampling was performed on the land elevation and crop cover product. This was carried out by taking a much larger simple random sample of 10 million transects, defining evenly spaced strata on the observed mean for each transect, then resampling evenly from each strata to give a mean-balanced sample. The sampling strategy of breeding birds in Catalonia was not designed or carried out by the authors of this study, but the sampling strategy is described henceforth to the best of the authors knowledge. First, a variable effort survey was carried out relying on the expertise of surveyors to judge the time and effort required to find all species expected in 385 10 x 10 km UTM squares. Second, 1-hour census in each of 3,077 systematically placed 1 × 1 km UTM squares was carried out, where all species observed were recorded. The results were then scaled to 385 10 x 10 km UTM squares. The second systematic census was then distributed for analysis and the first could be used to validate all species were found.
Data collection	Data collection was not carried out by the authors of this study, but the methodology is described briefly henceforth. The land elevation product was collected between 60°N and 58°S by NASA's Shuttle Radar Topography Mission. The mission utilised a spaceborne imaging radar-C/X-band synthetic aperture radar with additional C- and X-band antennas on a 60 meter mast. The additional antennas allowed assessment of phase difference between images and determination of distance with a single pass. The raw data containing voids due to water and mountain shadow was then void filled using interpolation by the Consortium of International Agricultural Research Centers Consortium for Spatial Information. The crop cover data was predicted between 78.25°N and 60°S by the Copernicus Global Land Service using vegetation data collected from the ESA PROBA-V satellite observations. The PROBA-V instrument collected images for four bands: blue (0.463 µm), red (0.655 µm), NIR (0.837 µm), and SWIR (1.603 µm).
Timing and spatial scale	Breeding birds in Catalonia collection: 1999–2002. Global land elevation above sea level: 2008. Global crop cover percentage: 2019.
Data exclusions	Breeding bird data in 100 10 x 10 km UTM grid cells on the border of the sampling area were removed due to reduced sampling effort, resulting in a sample size of 285.
Reproducibility	Code was tested on multiple machines. The experiment and sampling procedure were not repeated.
Randomization	There was no allocation to groups carried out by the authors of this study.

Blinding	Blinding was not carried out as there was no interaction between the researchers of this study and participants/objects/individuals of data collection.

Did the study involve field work? ☐ Yes ☒ No

Reporting for specific materials, systems and methods

We require information from authors about some types of materials, experimental systems and methods used in many studies. Here, indicate whether each material, system or method listed is relevant to your study. If you are not sure if a list item applies to your research, read the appropriate section before selecting a response.

Materials & experimental systems

n/a	Involved in the study
☒	☐ Antibodies
☒	☐ Eukaryotic cell lines
☒	☐ Palaeontology and archaeology
☒	☐ Animals and other organisms
☒	☐ Clinical data
☒	☐ Dual use research of concern
☒	☐ Plants

Methods

n/a	Involved in the study
☒	☐ ChIP-seq
☒	☐ Flow cytometry
☒	☐ MRI-based neuroimaging

Plants

Seed stocks	n/a
Novel plant genotypes	n/a
Authentication	n/a
